# Pharmacological mechanisms of traditional Chinese medicine metabolites in regulating Treg cells: an integrative pathway review

**DOI:** 10.3389/fphar.2025.1527421

**Published:** 2025-12-11

**Authors:** Chao Wang, Ming-Jie Liao, Yao Wu, Heng Lin, Zhen-Zhong Ye, Wen-Zhe Ma, Qing Yuan

**Affiliations:** 1 Public Center of Experimental Technology, The School of Basic Medical Sciences, Southwest Medical University, Luzhou, China; 2 Department of Thoracic Surgery, Affiliated Hospital of Southwest Medical University, Luzhou, China; 3 State Key Laboratory of Quality Research in Chinese Medicine and Faculty of Chinese Medicine, Macau University of Science and Technology, Macau, China; 4 Rehabilitation Medicine Department, Affiliated Hospital Of Southwest Medical University, Luzhou, China; 5 Department of Traditional Chinese Medicine, Affiliated Hospital of North Sichuan Medical College, Nanchong, China

**Keywords:** traditional Chinese medicine, regulatory T cells, Foxp3, immunomodulation, mechanisms

## Abstract

**Background:**

Regulatory T cells (Tregs), characterized by the transcription factor *Foxp3*, play a pivotal role in maintaining immune homeostasis, preventing autoimmunity, and contributing to tumor immune evasion. Traditional Chinese Medicine (TCM), with its long history of clinical application, exerts unique regulatory effects on immune responses. However, a comprehensive mechanistic synthesis of TCM-mediated Treg regulation remains lacking.

**Methods:**

We reviewed studies from PubMed up to August 2025, focusing on molecular, cellular, and microbiota-related mechanisms by which TCM modulates Tregs. Identified evidence was synthesized into four major mechanisms and further integrated into three regulatory axes.

**Results:**

TCM regulates Tregs through four mechanisms: (1) Foxp3 expression regulation mechanisms; (2) IL-2 receptor pathway mechanisms; (3) Regulation of other Treg surface molecules; and (4) Gut microbiota modulation mechanisms. These four mechanisms converge into three regulatory axes: the core execution axis (direct *Foxp3* control), the upstream regulatory axis (cytokine and receptor crosstalk), and the cross-boundary integration axis (gut microbiota–immune interactions).

**Conclusion:**

This review proposes an integrated framework that refines four regulatory mechanisms into three axes, highlighting the multi-layered and interconnected pathways through which TCM shapes Treg biology. This systems-level perspective provides a theoretical basis for developing TCM-derived strategies in immune-mediated diseases and cancer immunotherapy.

## 1 Introduction

Regulatory T cells (Tregs) are a specialized subset of *CD4* ^*+*^ T cells characterized by the expression of the master transcription factor *Foxp3* (Sakaguchi et al., 2008; Sakaguchi et al., 2010; Liston and Gray, 2014). These cells are defined by their potent immunosuppressive functions, which they mediate through the secretion of inhibitory cytokines direct cytolysis, inhibition of dendritic cell maturation, and metabolic disruption (Hatzioannou et al., 2021; Ou et al., 2023). Tregs play a crucial role in maintaining immune homeostasis, preventing autoimmune diseases, regulating inflammatory responses, and facilitating tumor immune evasion (Sakaguchi et al., 2020; Josefowicz et al., 2012). Their unique bidirectional function makes them potential therapeutic targets in various conditions, from autoimmune disorders to cancer (Mohr et al., 2019; Tanaka and Sakaguchi, 2017; Rezaei Kahmini et al., 2022; La, 2018; Li M. et al., 2020; Togashi et al., 2019; Esensten et al., 2018). Approaches aimed at either augmenting (Trotta et al., 2018; Zou et al., 2018) or reducing (Tanaka and Sakaguchi, 2017; Sugiyama et al., 2013) Treg numbers and modulating their suppressive activity represent novel therapeutic strategies already applied in clinical practice (Sakaguchi et al., 2020; Li C. et al., 2020; Liang et al., 2021).

As a natural product, Traditional Chinese Medicine (TCM) has demonstrated unique efficacy in the treatment of various diseases (Luo et al., 2019; Zhong et al., 2022; Chen et al., 2023). Numerous studies (Peng et al., 2022; Chen et al., 2019; Paik et al., 2019; Hoffman et al., 2020; Chen F. et al., 2021; Chen Y. et al., 2024) have demonstrated their ability to regulate various immune cells, including T cells (Ma et al., 2014), macrophages (Bamodu et al., 2019), dendritic cells (DCs) (Han et al., 2022) and natural killer (NK) cells (Lee et al., 2014). Like Tregs, these botanical drugs and their metabolites often display a dual regulatory capacity (Ma et al., 2013), either enhancing or suppressing immune responses depending on the specific disease context (Deng et al., 2022). However, while research has confirmed that TCM can modulate Tregs to alleviate autoimmune damage and enhance antitumor immune responses, a systematic and critical analysis of the specific pharmacological mechanisms by which their metabolites achieve this remains lacking.

Therefore, this review aims to provide a comprehensive and critically-assessed summary of the current evidence on how TCM botanical drugs and their metabolites modulate Tregs. In an era of precision medicine, understanding these specific regulatory mechanisms is crucial for developing more targeted and effective TCM-based immunotherapies. To achieve this, this review systematically integrates existing findings and proposes a hierarchical quad-mechanistic framework (master transcription factors, *IL-2* receptor pathways, surface molecules, and gut microbiota) and three interconnected regulatory axes, aiming to provide a clear theoretical basis for future TCM-based immunotherapy research.

## 2 Search strategy and study selection

To ensure a comprehensive and non-biased collection of relevant literature, this review adhered to a systematic search and selection strategy.

### 2.1 Search strategy

The international literature search was performed in PubMed. The final search was conducted up to August 2025. We employed a combination of Medical Subject Headings (MeSH) terms and free-text keywords covering three conceptual domains: (1) Traditional Chinese Medicine (TCM) Metabolites: *(“Traditional Chinese Medicine” OR “TCM” OR “Chinese herbal medicine” OR “botanical drug”OR “natural product”)*. (2) Target Immune Cells: *(“Regulatory T cells”OR “Tregs” OR “Foxp3”)*. (3) Mechanism and Disease: *(“Mechanism” OR “signaling pathway” OR “immunomodulation” OR “autoimmunity” OR “cancer” OR “inflammation” OR “gut microbiota”)*. The search was structured using Boolean operators (AND, OR) to ensure maximum sensitivity and relevance. A core search string in PubMed, for example, included: ((“TCM” OR “botanical drug” OR “natural product”) AND (“Treg” OR “regulatory T cells” OR “*Foxp3*”) AND (“signaling pathway” OR “mechanism”)).

### 2.2 Study selection process and data extraction

The final selection of included studies followed a rigorous three-step process. (1) Initial screening: two independent reviewers screened all titles and abstracts to remove duplicates and clearly irrelevant papers based on the exclusion criteria. (2) Full-text review: The same two reviewers independently assessed the full text of all potentially relevant articles. Discrepancies were resolved through discussion and consensus, or consultation with a third senior reviewer. (3) Final data collection (eligibility criteria): Only studies that explicitly elucidated the molecular signaling mechanisms underlying the regulation of Treg cells by TCM-derived metabolites were included for qualitative synthesis. A total of 82 studies met the inclusion criteria and were included in the qualitative analysis. For each included publication, the corresponding compound, experimental model type (animal, cellular, or human), dosing information, administration route, and mechanism of Treg regulation are summarized in Tables 1–3. The scientific rigor of each of the 82 included studies was critically appraised and assigned a rating (High/Moderate/Low Rigor). This assessment is presented in Supplementary Table S1. The appraisal criteria focused on: (1) Model Relevance (physiological context); (2) Controls and Completeness (clear dose-response and appropriate control groups); and (3) Mechanistic Depth (clear distinction between Treg phenotype and function reporting). This ensures that the foundation of our synthesis is transparently sourced and critically evaluated.

**TABLE 1:** TCMs exert therapeutic effects in immune-mediated autoinflammatory diseases by regulating Tregs.

Botanical drug medicine	Immune-mediated inflammatory diseases	Treg cell regulation	Species	Dose	Mechanism of action	Impact on anti-inflammatory factors	Key findings	References
Paeoniflorin	Inflammatory Bowel Disease	Increase	C57BL/6 mice	20 mg/kg/day in day 5 and day 10/	Modulates dendritic cell-mediated TH17/Treg balance, reduces inflammation	↑ IL-10,Foxp3 ↓ IL-2, IL-17	Paeoniflorin improves UC by modulating the TH17/Treg balance, enhancing anti-inflammatory cytokines	Zheng et al. (2020)
6-Gingerol		Increase	Male BALB/cmice	Low-dose group: 100 mg/kg. High-dose group: 250 mg/kg	Regulates Th17/Treg balance to reduce inflammation	↑ IL-10, ↓IL-17, IL-6	6-Gingerol alleviates colitis by balancing Th17/Treg and reducing both local and systemic inflammation	Sheng et al. (2020)
Total Glycosides of Peony		Increase	Male BALB/cmice	The low, medium and high dose groups of TGP 60, 120 and 240 mg/kg/day, respectively	Regulates IL-23/IL-17 axis and Th17/Treg balance	↑ IL-10, ↓IL-17A, IL-6, IL-23, TNF-α, IFN-γ, IL-9	Total glycosides of peony protect against IBD by regulating immune balance and reducing inflammation	Li et al. (2019)
Wu-Teng-Gao	Inflammatory Arthritis	Increase	SPF grade rats	high (0.45 g/paw), medium (0.3 g/paw), and low dose (0.15 g/paw)	Modulates Th17/Treg balance, reduces joint inflammation and cartilage destruction	↑ IL-10, TGF-β, ↓ IL-17, TNF-α, IL-1, IL-6	Wu-Teng-Gao alleviates joint inflammation by modulating the Th17/Treg balance	Yao et al. (2022)
Soufeng Sanjie Formula		Increase	Female DBA/1J mice	Low-dose group (183 mg/kg) High-dose group (550 mg/kg)	Inhibits Th17 cell differentiation, reduces IL-17A production	↑ IL-10,↓TNF-α, IL-6, IL-17A	Soufeng Sanjie formula reduces arthritis symptoms by restoring Th17/Treg balance	Hua et al. (2021)
Gancao Fuzi Decoction		Increase	Male DBA/1J mice	low-dose group (2.4 g/kg group) high-dose group (4.8 g/kg group)	Targets Foxp3 via miR-34a to regulate Th17/Treg imbalance	↑IL-10,↓(TNF-α), IL-1β, IL-6	Gancao Fuzi Decoction regulates Th17/Treg cell imbalance in rheumatoid arthritis by targeting Foxp3 via miR-34a	Zhao et al. (2023)
Zishen Tongluo Formula		Increase	Male DBA/1J mice (6–8 weeks)	0.84 g/mL ZTF, 0.22 g/mL YY, 0.43 g/mL QF, and 0.19 g/mL HT	Regulates Th17/Treg balance, reduces pro-inflammatory cytokines	↑ TGF-β,IL-2, IL-10,↓ IL-6, IL-23, IL-17,IL-21	Zishen Tongluo Formula alleviates arthritis by restoring Th17/Treg balance	Yang et al. (2020)
Quercetin		Increase	Female Wistar rats (150–170 g)	150 mg/kg	Activates HO-1 pathway, reduces oxidative stress and inflammation	↑ IL-10, TGF-β, ↓IL-17, IL-21	Quercetin alleviates arthritis by balancing Th17/Treg and activating anti-inflammatory HO-1 pathway	Yang et al. (2018)
Leonurine		Increase	Sprague Dawley (SD)rat	low-dose group (10 μM) and high-dose group (20 μM)	Inhibits TAZ expression to regulate Treg/Th17 balance	↑ IL-10, ↓IL-17,IL1β, TNF-α	Leonurine alleviates arthritis through Th17/Treg balance regulation via TAZ inhibition	Du et al. (2020)
Cinnamtannin D1		Increase	Male DBA/1 mice aged 6–8 weeks and BALB/c mice aged 6–7 weeks	20, 50 mg/kg/day	Inhibits AhR, reduces Th17 differentiation, promotes Treg activation	↑ IL-10, TGF-β,↓IL-17	Cinnamtannin D1 attenuates autoimmune arthritis by regulating Th17/Treg balance via AhR inhibition	Shi et al. (2020)
Curcumin and Curcuma longa Extract		Increase	—	—	Restores Treg/Th17 balance	↑TGF-β, IL-10,↓IL-6, TNF-α	Curcumin and Curcuma Longa extracts improve symptoms of arthritis by reducing inflammation and pain, with good safety profile across 29 RCTs involving 2,396 participants	Zeng et al. (2022)
Nanocurcumin	Ankylosing Spondylitis	Increase	PBMC	nanocurcumin capsules	Decreases IL-17, IL-23, RORγt mRNA expression	↑ IL-10, TGF-β,↓sIL-6	Nanocurcumin improves Treg cell responses and reduces inflammation in ankylosing spondylitis patients	Ahmadi et al. (2020)
Dihydroartemisinin	Systemic Lupus Erythematosus (SLE)	Increase	female BALB/c mice 6–8 weeks	100 mg/kg	Restores Treg/Th17 balance	↑ TGF-β,↓ IL-17, IL-6	Dihydroartemisinin restores the Treg/Th17 balance in lupus, alleviating disease symptoms	Chen Y. et al. (2021)
Total Glucosides of Paeony		Increase	PBMC	0, 62.5, 312.5 and 1,562.5 μg/mL	Increases Treg cells via FoxP3 demethylation	↑ IFN-γ, IL-2	Total glucosides of Paeony increase Treg cells and reduce lupus symptoms by FoxP3 demethylation	Zhao et al. (2012)
Xiaoying Daotan Decoction	Autoimmune Thyroiditis	Increase	female CBA/J mice	0.2 mL	Modulates Notch pathway to balance Treg/Th17	↑ Foxp3,IL-10, TGF-β,↓ IL-22, IL-17	Xiaoying Daotan Decoction regulates Treg/Th17 balance by Notch pathway to treat Hashimoto’s thyroiditis	Zhou et al. (2021)
Yanghe Decoction		Increase	Female Sprague-Dawley rats	5 g crude drug/kg and 15 g crude drug/kg	Regulates NLRP3 inflammasome, improves immune dysregulation	↑ IL-10, TGF-β,IL-35,↓IRORγt,L-17A,IL-21, IL-22	Yanghe Decoction improves immune dysregulation and NLRP3 inflammasome in autoimmune thyroiditis	Ma et al. (2021)
Artemisinin and Hydroxychloroquine	IgA Nephropathy	Increase	SPF male Sprague-Dawley rats	low dose (16.65 mg/kg) medium dose (33.33 mg/kg), high dose (66.66 mg/kg)	Regulates CD4^+^ T cells	↓IFN-γ, Foxp3	Combination of artemisinin and hydroxychloroquine suppresses CD4^+^ T cell differentiation in IgA nephropathy	Bai et al. (2019)
Hirudin		Increase	SPF male Sprague-Dawley rats	10 mg/kg	Inhibits fibrosis and inflammation, Reduces Treg/Th17 imbalance	↓IL-1b,IL-6, IL-18	Hirudin ameliorates IgA nephropathy by reducing fibrosis and inflammation	Deng et al. (2019)
Mangiferin	Lupus Nephritis	Increase	C57BL/6 mice	20 and 40 mg/kg	Increases Treg cells by suppressing mTOR signaling	↓IFN-γ,IL-6, TNF-α	Mangiferin attenuates lupus nephritis by inducing CD4+FoxP3+ regulatory T cells	Liang et al. (2018)
Baicalin		Increase	12-week-old female MRL-Fas(lpr) mice	200 mg/kg	Inhibits Tfh cells, expands Tfr cells	↑ Foxp3,TGF-β, IL-10	Baicalin ameliorates lupus by inhibiting Tfh cell differentiation and expanding Tfr cells	Yang J. et al. (2019)
Piperlongumine		Increase	11-week-old female MRL-Fas(lpr) mice	2.4 mg/kg	Regulates Treg/Th17 balance	↓ IL-6, IL-17, IL-23, TNF-α	Piperlongumine alleviates lupus nephritis by regulating the Treg/Th17 cell balance	Yao et al. (2014)

## 3 TCM exerts therapeutic effects on various diseases by regulating Tregs

### 3.1 Immune-mediated autoinflammatory diseases

In immune-mediated inflammatory diseases, TCM can modulate Tregs and increase the secretion of anti-inflammatory cytokines (Sakaguchi et al., 2020; Esensten et al., 2018) to restore the Th17 (T helper 17 cell)/Treg balance, thereby suppressing excessive immune responses and alleviating inflammation (Xie et al., 2022; Xu Y. et al., 2020). Paeoniflorin, a monoterpene glycoside extracted from the roots of Paeonia lactiflora, can significantly promote the differentiation of *CD4* ^*+*^ *CD25+Foxp3+* Tregs and suppress the production of Th17 cells, restoring the Th17/Treg balance for the treatment of inflammatory bowel disease (IBD). This leads to a marked reduction in histological scores in colitis models, revealing its potential anti-inflammatory mechanism (Zheng et al., 2020). Similarly, 6-gingerol, which is extracted from the rhizome of ginger, has been shown to reduce inflammation by modulating the Th17/Treg balance in a dextran sulfate sodium-induced colitis mouse model (Sheng et al., 2020). Additionally, total glycosides of peony (TGP) demonstrated significant efficacy in alleviating colitis induced by 2,4,6-trinitrobenzene-sulfonic acid, with higher doses providing effects comparable to sulfasalazine. These findings underscore the therapeutic potential of TGP in the management of IBD (Li et al., 2019).

In immune-mediated arthritis (IA), TCM also regulates immune responses by restoring the Th17/Treg balance, reducing joint inflammation, and preventing tissue damage (Li W. et al., 2023). In a collagen-induced arthritis mouse model, Wu Teng Gao can alleviate joint inflammation and bone destruction by increasing the number of Tregs and modulating the Th17/Treg balance (Yao et al., 2022). Other TCM compound formulations, such as Sou feng San Jie formula (Hua et al., 2021), Gan Cao Fu Zi decoction (Zhao et al., 2023), Zi Shen Tong Luo formula (Yang et al., 2020), as well as extracts like quercetin (Yang et al., 2018), leonurine (Du et al., 2020), and cinnamtannin D1 (Shi et al., 2020), have demonstrated anti-arthritis effects by rebalancing the Th17/Treg ratio. A meta-analysis evaluated the efficacy and safety of Curcumin and Curcuma longa extract in the treatment of arthritis, demonstrating significant improvements in arthritis symptoms and reductions in inflammation levels (Zeng et al., 2022). In patients with ankylosing spondylitis, curcumin significantly increases the number of Tregs, enhances the expression of transforming growth factor-β (*TGF-β*) and interleukin-10 (*IL-10*), and suppresses interleukin-6 (*IL-6*) levels (Ahmadi et al., 2020).

In the context of systemic lupus erythematosus (SLE), dihydroartemisinin (DHA), an extract isolated from the traditional Chinese botanical drug *Artemisia annua* L., has been shown to induce Treg differentiation, significantly increase the Treg ratio, and stimulate *TGF-β* secretion. When combined with prednisone, DHA exhibits a synergistic effect, enhancing therapeutic outcomes (Chen Y. et al., 2021). Additionally, TGP treatment significantly increased the proportion and number of Tregs among lupus *CD4* ^*+*^ T cells, indicating that TGP may inhibit autoimmunity in SLE patients by promoting Treg differentiation (Zhao et al., 2012).

In autoimmune thyroiditis (AIT), TCM has demonstrated effective therapeutic effects on AIT (Zhou et al., 2024). Xiao Ying Dao tan decoction, which consists of herba Xia KuCao, Fritillaria (Tubeimu), and Bupleurum (Chai Hu), can effectively downregulate Notch protein expression in Hashimoto’s thyroiditis mouse models and thyroiditis cells while upregulating Treg cytokines and downregulating Th17 cytokines to treat Hashimoto’s thyroiditis (Zhou et al., 2021). Yang He decoction (composed of Radix Rehmanniae praeparata, Cortex Cinnamomi, Ephedra sinica stapf, Semen brassicae, Zingiber offcinale Rose, Radix Rhizoma glycyrrhizae, and Colla cornus cervi) alleviates experimental autoimmune thyroiditis in rats by restoring the Th17/Treg imbalance and improving the *NLRP3* inflammasome (Ma et al., 2021).

In immunoglobulin A nephropathy (IgAN), the combination of artemisinin and hydroxychloroquine enhances Treg differentiation and reduces IgA immune complex and complement 3 deposition, which significantly improves renal dysfunction (Bai et al., 2019). In a bovine gamma-globulin-induced IgAN mouse model, hirudin reversed the BGG-induced reduction of *CD4* ^*+*^ *CD25* ^*+*^ *Foxp3* ^*+*^ Tregs, thereby maintaining immune homeostasis to prevent IgAN (56). In lupus nephritis (LN), mangiferin increases the proportion of *CD4* ^*+*^ *Foxp3* ^*+*^ Tregs and inhibits the *mTOR/p70S6K* pathway in FasL-deficient B6/gld mice, serving as a therapeutic agent for LN (57). Both baicalin (Yang J. et al., 2019) and piperlongumine (Yao et al., 2014) promote *CD4* ^*+*^ *Foxp3* ^*+*^ Tregs accumulation, thereby alleviating LN.

For other immune-related diseases, such as primary Sjögren’s syndrome, systemic sclerosis, and psoriasis, TCM has demonstrated therapeutic effects by reducing Th17 cell levels and promoting Treg generation (Xu Y. et al., 2020; Jun et al., 2021).

In summary, TCM can alleviate autoimmune inflammation by promoting Treg differentiation, restoring the Th17/Treg balance, and enhancing Treg secretion of anti-inflammatory cytokines (Table 1). Moreover, these studies have expanded the application of TCM in treating diseases such as IBD, IA, and SLE. However, what role do TCMs play in treating tumors? How do they affect Tregs?

### 3.2 Tumors

In the TME, conventional T cells in the blood can be induced to differentiate into Tregs, leading to immunosuppression—one of the key mechanisms of tumor immune evasion (Sakaguchi et al., 2020; Toker and Ohashi, 2019; Campbell and Rudensky, 2020). Therefore, strategies aimed at reducing Treg differentiation or inhibiting Treg function offer promising therapeutic avenues for cancer treatment. TCM has also demonstrated significant antitumor effects in various cancers by modulating Treg activity (Tanaka and Sakaguchi, 2017; Zeng et al., 2020).

#### 3.2.1 Lung cancer

Lung cancer is the most common cancer worldwide and threatens human life and health (Bray et al., 2024). In lung cancer treatment, TCM has demonstrated favorable therapeutic outcomes and synergistic effects, whether used in standard therapy, combination chemotherapy, targeted therapy, or immunotherapy. For example, the Fei Yan Ning Decoction has been found to enhance antitumor immune responses by reducing the proportion of *CD4* ^*+*^ *CD25* ^*+*^ regulatory T cells and downregulating *Foxp3* mRNA expression in mice bearing Lewis lung carcinoma (Guo et al., 2012). Fu Zheng Fan Gai Pill, when combined with the chemotherapeutic agent cyclophosphamide, significantly reduces the proportion of *CD4+IL-17+* Th17 and *CD4* ^*+*^ *CD25+Foxp3+* Tregs in the spleen and metastatic lesions of mice with Lewis lung cancer. Additionally, it inhibits *Foxp3* and *RORγt* mRNA expression, thereby markedly reducing cancer growth and metastasis by suppressing the *SOCS/JAK-STAT* pathway and inflammatory cytokine responses (Liu et al., 2014). Both luteolin and apigenin significantly inhibit the proliferation of KRAS-mutant lung cancer cells and downregulate *IFN-γ*-induced *PD-L1* expression, showing synergistic effects when combined with *PD-1* inhibitors. In lung cancer mouse models treated with apigenin, the proportion of Tregs in the spleen and blood is reduced, further contributing to the antitumor activity of apigenin (Jiang et al., 2021). The aqueous extract of Taxus chinensis var. mairei, when combined with anti-PD-1 drugs in a mouse model of Lewis lung cancer, reduces the ratio of *CD25+Foxp3+* Tregs and enhances synergistic effects by promoting antitumor immune responses (Dai et al., 2022). Berberine significantly inhibits the activation of myeloid-derived suppressor cells (MDSCs) and Tregs in the TME of a lung cancer mouse model, enhancing the immune activity of tumor-infiltrating T cells and shifting the immune microenvironment from immunosuppression to immune activation. Additionally, berberine reduces *PD-L1* expression in cancer cells by inhibiting *CSN5* deubiquitination, resulting in significant antitumor effects in Lewis tumor-bearing mice (Liu et al., 2020).

#### 3.2.2 Breast cancer

Breast cancer is a leading cause of death among women worldwide, and its treatment has garnered significant attention from TCM researchers (Bray et al., 2024). Studies have indicated that artemisinin inhibits the growth of 4T1 breast cancer cells *in vivo* by enhancing T-cell activation and inhibiting the immunosuppressive activity of Tregs and MDSCs within the tumor (Cao et al., 2019). Oridonin suppresses Treg differentiation and attenuates their immunosuppressive function by reducing *TGF-β* receptor protein levels, thereby delaying the progression of triple-negative breast cancer. Moreover, oridonin exhibits synergistic effects with anti-*PD-1* therapy, resulting in enhanced tumor regression when used in combination (Guo et al., 2020). Another formulation with synergistic anticancer effects is Aiduqing formula (ADQ), which is composed of oldenlandia diffusa, curcuma zedoaria, astragalus membranaceus and glycyrrhiza uralensis fisch. This formula works synergistically with paclitaxel to inhibit the development of breast cancer (Wang et al., 2018). Further research has shown that ADQ reshapes the immunosuppressive TME of breast cancer and inhibits breast cancer metastasis by reducing Treg differentiation and infiltration through suppression of the *NF-κB/Foxp3* pathway. Additionally, ADQ has no significant hepatotoxicity, nephrotoxicity, or hematotoxicity, making it a promising treatment option for breast cancer (Li et al., 2021).

#### 3.2.3 Colon cancer

As one of the top three malignant tumors in terms of global mortality, colon cancer has shown significant treatment responses to the regulatory effects of TCM(64). The botanical drug formula Yi-Yi-Fu-Zi-Bai-Jiang-San (Yi-Yi-ren, Fu-Zi, Bai-Jiang-Cao) reduces the expression of *IL-6*, *CCXL13*, and *IL-10*, thus mediating immune cell regulation. It also modulates the natural gut microbiota in mice and inhibits the proliferation of *CD4* ^*+*^ *CD25+Foxp3+* Tregs that accumulate in the intestinal and mesenteric lymph nodes, indirectly suppressing the growth of colorectal cancer cells (Sui et al., 2020). Baicalein enhances the antitumor effects of immunotherapeutic agents by eliminating *TNFR2-*related Treg activity. The primary mechanism involves disrupting the *TNF-TNFR2* interaction and inhibiting the phosphorylation of the downstream signaling component *p38MAPK*. In a CT26 colon cancer mouse model, baicalein treatment significantly improved the efficacy of CpG oligodeoxynucleotide tumor immunotherapy (Chen S. et al., 2022). Shenling Baizhu decoction also increases the abundance of the gut microbiota and modulates the tumor immune microenvironment by increasing the number of M1 macrophages while reducing the number of M2 macrophages and Tregs, thereby increasing the efficacy of tislelizumab and producing a synergistic antitumor effect (Deng et al., 2024).

#### 3.2.4 Liver cancer

Liver cancer often has a hidden onset and is typically diagnosed at a late stage (Bray et al., 2024). Various TCMs have been shown to enhance antitumor immunity by m odulating Tregs and inhibiting the progression of liver cancer. Ganoderma lucidum polysaccharides can inhibit the expression of Notch1 and Foxp3 by upregulating the expression of *miR-125b*, thereby suppressing Treg function and inhibiting the growth of liver cancer cells (Li et al., 2015). Astragalus polysaccharide (APS), the primary active extract of Astragalus, can inhibit the growth and proliferation of *CD4* ^+^ *CD25* ^*high*^ Tregs and block their migration by suppressing *SDF-1* or its receptor via the *CXCR4/CXCL12* pathway. APS exerts antitumor effects by restoring the cytokine balance in the TME and reducing the immunosuppressive function of Tregs via *Foxp3* mRNA inhibition (Li et al., 2012). Hydroxysafflor Yellow A (HSYA) demonstrated significant anti-hepatocarcinoma immunomodulatory activity. In the Hepa1-6 mouse model, HSYA could downregulate the *FOXP3+* level, effectively suppressing the Treg cell ratio. Crucially, HSYA inhibited tumor growth while, in contrast to Cisplatin, did not cause body weight loss, highlighting its potential advantage in terms of lower toxicity (Wang Y. et al., 2024). Radix glycyrrhizae polysaccharide can inhibit the growth of hepatocellular carcinoma by downregulating Tregs in a liver cancer model mice and reducing the serum levels of *IL-10* and *TGF-β* while upregulating the expression of the cytokines *IL-2* and *IL-12p70* (He et al., 2011). Similarly, Scutellaria barbata D. Don extract (SBE) suppressed liver cancer cell proliferation *in vitro* in a dose-dependent manner and inhibited tumor growth in H22 liver cancer-bearing mice. This effect is associated with SBE reducing Treg numbers and lowering the expression of *IL-10, TGF-β,* and *IL-17A*, while increasing *IL-2* and *IFN-γ* levels in the serum (Kan et al., 2017). Additionally, Dahuang Zhechong Pill treatment in a mouse model of *in situ* liver cancer increased the number of Th1 cells in the peripheral blood and spleen, with increased *IFN-γ* secretion activating CD8 T cells and inhibiting Treg production, thereby suppressing hepatocellular carcinoma growth (Chen T. T. et al., 2022).

#### 3.2.5 Other tumors

In addition to several high-incidence tumors, TCMs exert anticancer effects on various other tumors by regulating Tregs. In gastric cancer, modified Bu Zhong Yi Qi decoction reduced the proportions of *CD8+PD-1+* T cells and *PD-1+* Tregs in the peripheral blood of gastric cancer model mice through the *PI3K/AKT* pathway. It acts synergistically with 5-fluorouracil to inhibit gastric cancer progression and, as adjuvant therapy postchemotherapy, significantly prolongs the survival time of gastric cancer patients (Xu R. et al., 2020). In bladder cancer, lentinan is an isolated botanical drug metabolite that induces macrophage activation in a bladder cancer mouse model, promoting the proliferation of *CD4* ^*+*^ and *CD8* ^*+*^ T cells while upregulating the expression of *IFN-γ* and *IL-2*. Concurrently, it inhibited the proliferation of MDSCs and Tregs, as well as the expression of anti-inflammatory cytokines *IL-10* and *TGF-β*. In combination with gemcitabine, lentinan activated immunity and had a synergistic effect on the suppression of bladder tumor growth in a mouse model (Sun et al., 2020). In cervical cancer, artesunate inhibits *in situ* tumor growth and exerts antitumor effects by suppressing *PGE2* production in CaSki and HeLa cells and downregulating *Foxp3* expression in T cells (Zhang et al., 2014). In melanoma, triptolide inhibits melanoma growth by suppressing the generation of regulatory T cells and the production of several cytokines, such as *IL-10*, *TGF-β*, and vascular endothelial growth factor (Liu B. et al., 2013). In head and neck squamous cell carcinoma, curcumin restored the cytotoxic function of effector T cells by modulating the expression of *PD-1* and *TIM-3* on *CD4+*or *CD8* ^*+*^ T cells and *CD4* ^*+*^CD2*5+Foxp3+* Tregs, promoting the expression of *IFN-γ* and granzyme B to exert its anticancer effects. Additionally, curcumin, in combination with *PD-L1* antibodies, enhanced the cytotoxic activity of *CD8* ^*+*^ T cells (Liu et al., 2021).

In summary, TCMs can suppress the differentiation of Tregs within the TME, attenuate their immunosuppressive effects, and enhance the body’s anticancer immune response. Moreover, TCM exhibits synergistic effects when combined with chemotherapy, targeted therapies, and immunotherapeutic agents across various types of tumors. (Table 2).

**TABLE 2:** TCMs exert therapeutic effects in tumors by regulating Tregs.

Botanical drug medicine	Immune-mediated inflammatory diseases	Treg cell regulation	Species/Cell line	Dose/Concentration	Mechanism of action	Impact on anti-inflammatory factors	Key findings	References
Feiyanning Decoction	Lung Cancer	Decrease	C57BL/6 mice and Lewi lung cancer cells	—	Reduces CD4^+^CD25^+^ Treg cell proportion, downregulates Foxp3 mRNA expression	—	Feiyanning Decoction enhances antitumor immunity by decreasing regulatory T cell ratios and FoxP3 expression	Guo et al. (2012)
FuZheng FangAi Pill		Decrease	6–8 weeks old male C57BL/6 mice	6.5 g	Inhibits Treg cell proportions, suppresses Foxp3 expression by SOCS3/JAK-STAT pathway	↑ IFN-γ,IL-17, IL-23,↓ IL-6, TGF-β	Inhibits tumor growth and metastasis	Liu et al. (2014)
Apigenin and luteolin		Decrease	Normal lung fibroblast cell line CCD-19Lu, human bronchial epithelial cell line BEAS-2B,KRAS-mutant human lung cell lines H358, H460, H2122, and A549	0, 10, 20, 30, 40, 50 μM	Downregulates IFN-γ-induced PD-L1 via suppression of STAT3 phosphorylation	—	Luteolin and apigenin significantly inhibit lung cancer growth, induce apoptosis, and show synergistic effects with PD-1 blockade	Jiang et al. (2021)
Aqueous extract of Taxus chinensis var. Mairei		Decrease	Mouse NSCLC cell line (HCC827-EGFR 19del G746-A750, LLC), human monocytic cell line THP-1, and mouse RAW264.7 macrophages Female C57BL/6 mice	(Cells) 0.13 or 1 mg/mL (Mice) 1.2 g/mL AETC	Regulates the tumor immune microenvironment, reduces tumor immune evasion	—	The aqueous extract improves the immune response against NSCLC and enhances the effects of immune checkpoint inhibitors	Dai et al. (2022)
Berberine		Decrease	NSCLC cell lines A549, H157, H358, H460, H1299, H1975, Lewis cells, and Jurkat (E6-1) cells. 8-week-old female C57BL/6 mice and BALB/c- nude mice	0, 2, 4, 8, 16, and 32 mg/kg BBR	Inhibits CSN5-mediated deubiquitination of PD-L1, enhancing PD-L1 degradation	—	Berberine enhances antitumor immunity by inhibiting PD-L1 expression through inhibiting the deubiquitination activity of CSN5	Liu et al. (2020)
Artemisinin	Breast Cancer	Decrease	6-8 female Balb/c mice	100 mg/kg)	Promotes T cell activation, inhibits Treg and MDSC	↑ T-bet, IFN-γ, , TNF-α,↓TGF-β	Artemisinin enhances anti-tumor immune response *in vitro* and *in vivo*, showing potential anti-cancer effects	Cao et al. (2019)
Oridonin		Decrease	Murine 4T1 breast cells and human MDA-MB-231 breast cancer cells/Female BALB/c mice and thymus-deficient BALB/c nude mice (6–8 weeks old)	5 mg/kg	Inhibits Treg differentiation and immunosuppressive ability by suppresses TGF-β receptor signaling	↓TGF-β 1, IL-10	Oridonin inhibits 4T1 tumor growth by suppressing Treg differentiation via the TGF-β receptor	Guo et al. (2020)
Aiduqing Formula		Decrease	Human breast cancer cell lines MDA-MB-231 and MCF-7	100 mg/kg	Remodeling the immunosuppressive TME of breast cancer and reducing Treg differentiation by inhibiting the NF-κB/FOXP3 pathway	—	Aiduqing Formula inhibits breast cancer metastasis by suppressing Treg cell infiltration	(Wang et al., 2018), (Li et al., 2021)
Yi-Yi-Fu-Zi-Bai-Jiang-San (YYFZBJS)	Colon Cancer	Decrease	Human colorectal adenocarcinoma HCT116 cells and mouse colorectal adenocarcinoma MC-38 cells C57BL/6	3.825 g/kg、7.65 g/kg and 15.3 g/kg	Modulates immune cell regulation, inhibits Treg cell proliferation, Remodels gut microbiota	↓IL-6, CCXL13, IL-10	YYFZBJS ameliorates colorectal cancer progression by modifying gut microbiota and reducing Treg cells	Sui et al. (2020)
Scutellarin		Decrease	Female wild-type (WT) C57BL/6J and Balb/c mice (8–12 weeks) WEHI 164 clone 13 cell line (WEHI-13VAR) and CT26 colon cancer cell line	25 mg/kg and 50 mg/kg	Reduces TNFR2-expressing CD4+Foxp3+ regulatory T cells	—	Scutellarin enhances anti-tumor immune responses by reducing TNFR2-expressing CD4+Foxp3+ regulatory T cells	Chen S. et al. (2022)
Shenling Baizhu Decoction (SLBZD)		Decrease	Male BALB/c-Hpd1 mice HCT116 cells	1 g/day (0.5 g/ml)	Influences gut flora and tumor microenvironment	—	SLBZD synergizes with Tirelizumab in colorectal cancer by balancing colon flora and immune responses	Deng et al. (2024)
Ganoderma Lucidum Polysaccharide Extract	Liver Cancer	Decrease	Mouse hepatocellular carcinoma H22 cell line and normal liver cell line L-02	10 mg/kg, 50 mg/kg, 100 mg/kg, and 200 mg/kg	Downregulates regulatory T cells accumulation and function by inducing microRNA-125b	↑ IL-2	Inhibits hepatocellular carcinoma growth by downregulating regulatory T cells accumulation and function through microRNA-125b induction	Li et al. (2015)
Astragalus Polysaccharides		Decrease	Human	–	Inhibits Treg cell growth and proliferation, suppresses CXCR4/CXCL12 pathway	↓ IL-10	Inhibits functions of CD4^+^CD25 high Treg cells in the tumor microenvironment of human hepatocellular carcinoma	Li et al. (2012)
Radix Glycyrrhizae polysaccharide		Decrease	Female BALB/c mice, Mouse H22 hepatocellular carcinoma cells	250 mg/kg/d	Downregulates Treg cells and upregulates Th1/Th2 cytokine ratio	↑ IL-2, IL-12p70,↓IL-10, TGF-β	Induces downregulation of Treg cells and upregulation of Th1/Th2 cytokine ratio in H22 hepatocarcinoma-bearing mice	He et al. (2011)
Scutellaria Barbata D. Don Extract		Decrease	Human hepatoma cell line (HepG2) and mouse hepatoma cell line (H22) Male BALB/c mice (18–22 g)	50, 100, and 150 mg/kg/day	Downregulates Treg cells and manipulates Th1/Th17 immune response	↓IL-10, TGF-β, , IL-17A,↑ IL-2, IFN-γ	Inhibits tumor growth by down-regulating Treg cells and manipulating Th1/Th17 immune response in hepatoma H22-bearing mice	Kan et al. (2017)
DaHuang ZheChong Pills		Decrease	Hepa1-6 cells C57BL/6 mice	2.4 g/kg and 1.2 g/kg	Reverses Treg/Th1 balance	↑ IFN-γ	The pills inhibit liver cancer growth by reversing Treg/Th1 balance	Chen T. T. et al. (2022)
Modified Bu-Zhong-Yi-Qi Decoction	Gastric Cancer	Decrease	Mouse gastric cancer cell line MFC cells, C57BL/6	2.03 g/mL,0.2 mL/10 g	Reduces PD-1 T cell and PD-1 Treg cell proportions by PI3K/AKT pathway	—	Inhibits gastric cancer progression by enhancing PD-1/PD-L1-dependent T cell immunization when combined with 5 Fluorouracile	Xu Y. et al. (2020)
Lentinan	Bladder Cancer	Decrease	male C57BL/6 mice; Mouse bladder cancer cell line MB49	4 mg/kg LNT, 40 mg/kg GEM 40 mg/kg LNT 40 mg/kg GEM	Induces macrophage activation, promotes CD4 and CD8 T cell proliferation and IFN-γ and IL-2 expression, inhibits differentiation of MDSCs and Tregs, and expression of IL-10 and TGF-β	↓IL-10, TGF-β	Inhibits tumor progression by modulating the immune response in a mouse model of bladder cancer	Sun et al. (2020)
Artesunate	Cervical Cancer	Decrease	Human cervical cancer cell lines CaSki and HeLa, human endocervical cell line H8, and T lymphocyte line Jurkat, Peripheral blood samples were obtained from 60 cervical cancer patients and 30 healthy volunteers; C57BL/6 female mice	10 mg/kg 50 mg/kg 100 mg/kg	Inhibits PGE2 production and Foxp3 expression	—	Exerts an anti-immunosuppressive effect on cervical cancer by inhibiting PGE2 production and Foxp3 expression	Zhang et al. (2014)
Triptolide	Melanoma	Decrease	Mouse B16-F10 melanoma cell line Pathogen-free C57BL/6 male mice, 6–8 weeks old	TH: 0.3 mg/(kg·d) TL:0.15 mg/(kg·d)	Inhibits Treg cell generation and pro-inflammatory cytokines	↓IL-10, TGF-β, VEGF	reduces Treg cells and various inflammatory cytokines in melanoma	Liu B. et al. (2013)
Curcumin	Head and Neck Squamous Cell Carcinoma (HNSCC)	Decrease	Human HNSCC cell lines SNU1076 (larynx), SNU1041 (hypopharynx), and FaDu (hypopharynx), SCC15 (oral tongue),and Six-week-old male nude mice	50 mg/kg	Regulates PD-1 and TIM-3 expression on T cells	↑IFN-γ, Granzyme B	Curcumin impacts immune checkpoint modulation and T cell functionality in cancer	Liu et al. (2021)

### 3.3 Other diseases

TCM exerts its therapeutic effects through the modulation of Tregs not only in autoimmune diseases and tumors, but also in other diseases. In metabolic diseases such as atherosclerosis, Bu Yang Huan Wu Decoction (BYHWD) promoted Treg differentiation, restored the immune balance among *CD4* ^*+*^ T cells, regulated lipid metabolism, and inhibited inflammatory responses, thereby demonstrating the potential to increase plaque stability (Chen S. et al., 2021). Similarly, glycyrrhizin increased the expression of *IL-10* and *IL-2*, and enhanced *STAT5* phosphorylation in Tregs, which improved lipid metabolism abnormalities in *Apoe−/−* mice and inhibited vascular inflammation, potentially alleviating atherosclerotic lesions (Ding et al., 2018). In hepatitis, Astragalus polysaccharide (APS), extracted from the roots of the traditional botanical drug medicine Astragalus, significantly increased the production of antigen-specific antibodies, T-cell proliferation, and cytotoxic T lymphocyte activity when co-administered with recombinant hepatitis B surface antigen. Moreover, it reduced the expression of *TGF-β* and the proportion of *CD4* ^*+*^ *CD25+Foxp3+* Tregs, thereby enhancing both humoral and cellular immune responses to hepatitis B surface antigen vaccination. This makes APS an effective adjuvant for hepatitis B subunit vaccines (Du et al., 2011). Water-extractable polysaccharides of Cistanche deserticola (WPCD) significantly upregulated *IgG, IgG1, and IgG2a* levels and enhanced the proliferation of T cells and B cells. WPCD also increased the production of *IFN-γ* and *IL-4* in *CD4+T* cells, as well as the expression of *IFN-γ* in *CD8+*T cells, while elevating *CD40* and *CD80* expression in splenic DCs and decreasing Tregs frequencies. Additionally, the WPCD activated DCs through the *TLR4* signaling pathway, thereby promoting both humoral and cellular immune responses and establishing it as a safe and effective vaccine adjuvant (Zhang et al., 2018). In stroke, Ginkgo biloba extract promoted the differentiation of *CD4+T* cells into Tregs by inhibiting HK2-mediated glycolysis. It increased the expression of the Treg transcription factor *Foxp3* and the cytokine *IL-10*, while decreasing the expression of the Th17 transcription factor *RORγt* and the Th17-specific cytokine *IL-17*. This regulation of the Th17/Treg balance improved ischemia/reperfusion injury in mice (Hui et al., 2023). In asthma, Ephedrae herba polysaccharides regulate the imbalance of Th1/Th2 and Th17/Treg, jointly inhibiting inflammation, apoptosis, and reactive oxygen species production in ovalbumin-induced asthmatic rats (Zhang et al., 2022). In pneumonia, an botanical drug formula containing eight plants, compound 511, modulates the balance of Th1/Th2 and Th17/Treg through the *PI3K/AKT/mTOR* signaling pathway. It reduces *Foxp3* and *GATA3* mRNA levels and increases *STAT3* and *T-bet* mRNA expression in the spleen, improving immune function and reducing lung inflammation caused by methicillin-resistant *Staphylococcus aureus* (Li Z. et al., 2023). In lung injury, Jiawei Maxing Shigan Tang (JMST) reduced the number of Tregs in lung tissue and alleviated the degree of pulmonary fibrosis. After intervention with JMST, the expression of *Smad2/3, p-Smad2/3, Smad4, TGF-β1, vimentin,* and *α-SMA* was significantly downregulated, whereas the expression of *E-cadherin* was upregulated. JMST alleviated radiation-induced lung injury by inhibiting epithelial-mesenchymal transition (EMT) through the *TGF-β1/Smad* pathway mediated by Tregs (Wang M. et al., 2024) (Table 3).

**TABLE 3:** TCMs exert therapeutic effects in other diseases by regulating Tregs.

Botanical drug medicine	Immune-mediated inflammatory diseases	Treg cell regulation	Species/Cell line	Dose/Concentration	Mechanism of action	Impact on anti-inflammatory factors	Key findings	References
BuYang HuanWu Decoction	Atherosclerosis	Increase	Six-week-old male ApoE−/− mice on a C57BL/6 genetic background and male wild-type C57BL/6 mice	5 g/kg 10 g/kg 20 g/kg	Regulates TGF-β/Smad2 pathway to promote differentiation of regulatory T cells	↑ TGF-β,↓IL-6、IFN-γ	Buyang Huanwu Decoction ameliorates atherosclerosis by regulating TGF-β/Smad2 to promote Treg cells	Chen Y. et al. (2021)
Glycyrrhizin		Increase	8-week-old male C57BL/6	50 mg/kg	Inhibits HMGB1 to improve lipid metabolism and increases Treg cell STAT5 phosphorylation reduces inflammatory	↑IL-10,IL-2,↓IL-17A,IL-6	Glycyrrhizin improves lipid metabolism and reduces vascular inflammation in atherosclerosis	Ding et al. (2018)
Astragalus Polysaccharides	Hepatitis B	Decrease	BALB/c mice	500 μg	Inhibits Treg cell growth and proliferation	↓TGF-β	Enhances immune responses by inhibiting TGF-β expression and frequency of regulatory T cells	Du et al. (2011)
Water-extractable polysaccharides of Cistanche deserticola		Decrease	8–10 week old female C57BL/6, BALB/c or ICR mice	0.01, 0.02, 0.05, 0.1 and 0.2 mg/mL	Activated dendritic cells induce humoral and cellular immunity through activation of TLR4 signaling pathway	—	Cistanche polysaccharides show immunostimulatory effects, acting as an adjuvant in vaccines	Zhang et al. (2018)
Ginkgo biloba extract	Ischemic Stroke	Increase	Male C57BL/6J mice	50 mg/kg 100 mg/kg 60 mg/kg	Promotes Treg differentiation via inhibition of HIF-1α/HK2 pathway	↑ IL-10	Ameliorates ischemic stroke by promoting TREG differentiation via inhibition of HIF-1α/HK2 pathway	Hui et al. (2023)
Ephedrae Herba polysaccharides	Asthma	Increase	rat	137.71 mg/kg/day and 275.42 mg/kg/day	Regulates Th1/Th2 and Th17/Treg cell immune imbalance	↑ IL-10,TGF-β1,IL-6	Inhibits inflammation of ovalbumin-induced asthma by regulating Th1/Th2 and Th17/Treg cell immune imbalance	Zhang et al. (2022)
Compound 511	Pneumonia	Decrease	C57BL/6 mice	3 g/kg,6 g/kg, 12 g/kg	regulates Th1/Th2 and Th17/Treg cell balance by RPI3K/AKT/mTOR pathway	↓IL-1β 、 TNF-α,IL-6	Ameliorates MRSA-induced lung injury by attenuating morphine-induced immunosuppression via PI3K/AKT/mTOR pathway	Li Z. et al. (2023)
Jiawei Maxing Shigan Tang	Lung injury	Decrease	Male Sprague-Dawley (SD) rat	1.25 g/kg	TGF-β1/Smad signaling pathway mediated by regulatory T cells	↓ TGF-β1	Alleviates radiation-induced lung injury via TGF-β1/Smad signaling pathway mediated by regulatory T cells	Wang Y. et al. (2024)

These findings suggest that various TCMs can modulate the quantity and function of Tregs through distinct regulatory mechanisms, providing therapeutic benefits across various diseases and effectively improving pathological conditions. Notably, the therapeutic effects of TCMs on the above diseases—whether autoimmune, neoplastic, or metabolic—are primarily achieved by modulating Treg cells. However, the specific ways in which TCMs regulate Treg cells (e.g., affecting their generation or modifying their function) remain to be systematically clarified. The following section will first focus on the two core aspects of TCM-mediated Treg regulation: the modulation of Treg generation and the regulation of Treg function, laying a foundation for further exploration of the underlying molecular mechanisms.

## 4 Pharmacological mechanisms of TCM metabolites in regulating Treg cells

It is evident from the literature summarized in the preceding sections that despite the chemical diversity of these metabolites, their regulatory effects primarily converge on two distinct aspects.

### 4.1 Phenotypic regulation (generation/number)

This involves controlling the differentiation, proliferation, and ultimately the number (frequency) of *CD4* ^*+*^ *CD25+Foxp3+*Treg cells, which is primarily driven by the regulation of the master transcription factor *Foxp3*. (1) Promotion (for immune-mediated inflammatory diseases): botanical drug(s) such as paeoniflorin (Zheng et al., 2020), curcumin (Zeng et al., 2022), mangiferin (Liang et al., 2018), Yun Nan Bai Yao (Ren et al., 2022). Licorice (Guo et al., 2015), ECa 233 (a standardized extract of *C. asiatica*) (Tawinwung et al., 2021), compound small peptide of Chinese medicine (Cui et al., 2023), promote *Foxp3* expression and Treg proliferation. (2) Inhibition (for Tumor-Related Diseases): Conversely, metabolite(s) such as baicalin (Yang J. et al., 2019), berberine (Liu et al., 2020), and the Aiduqing formula (Li et al., 2021), Astragalus polysaccharides (Du et al., 2011), Cistanche deserticola (Zhang et al., 2018), compound 511 (Li Z. et al., 2023), and curcumin (Liao et al., 2018; Yang et al., 2008) inhibit *Foxp3* expression and Treg proliferation.

### 4.2 Functional regulation (competence)

This refers to the adjustment of the Treg suppressive capacity, typically evidenced by the secretion of inhibitory cytokines. Firstly, the Treg suppressive function fundamentally relies on the stable expression of Foxp3 as discussed above (Sakaguchi et al., 2010; Hori et al., 2003). Secondly, it depends on inhibitory cytokines, especially *IL-10* and *TGF-β*, which play a key role in suppressing inflammatory responses and promoting immune tolerance (Li C. et al., 2020; Akdis and Akdis, 2014; Josefowicz et al., 2012). Consequently, studies on TCM-mediated Treg functional regulation frequently examine the expression levels of *Foxp3, IL-10,* and *TGF-β*, which are often correlated. (1) Promotion of Cytokine Secretion: TCM metabolite(s) that promote *IL-10* and *TGF-β* secretion include 6-Gingerol (Sheng et al., 2020), Wu Teng Gao (Yao et al., 2022), Soufeng Sanjie Formula (Hua et al. 2021), quercetin (Yang et al., 2018). (2) Inhibition of Cytokine Secretion: Conversely, metabolite like apigenin (Jiang et al., 2021), radix glycyrrhizae polysaccharide (He et al., 2011), and astragalus polysaccharides (Du et al., 2011), an cause Treg cells to reduce *TGF-β* and *IL-10* secretion, thereby attenuating Treg suppressive function. (Table 4).

**TABLE 4:** Regulation of Treg cell differentiation and cytokines by different TCMs.

Category of TCM	Promote Treg differentiation and cytokine release	Inhibit Treg differentiation and cytokine release
Chinese Medicine monomers	Paeoniflorin, 6-Gingerol, Quercetin, Leonurine, Cinnamtannin D1, Dihydroartemisinin, Hirudin, Mangiferin, Baicalin, Piperlongumine, Glycyrrhizin, Nanocurcumin, Curcumin	Apigenin, luteolin, Berberine, Artemisinin, Oridonin, Scutellarin, Curcumin, Triptolide, Artesunate
Chinese botanical drug extracts	Total Glycosides of Peony, Curcuma longa Extract, Ginkgo biloba extract, Ephedrae Herba polysaccharides	Aqueous extract of Taxus chinensis var. Mairei, Ganoderma Lucidum Polysaccharide Extract, Astragalus Polysaccharides, Radix Glycyrrhizae polysaccharide, Scutellaria Barbata D. Don Extract, Water-extractable polysaccharides of Cistanche deserticola, Lentinan
Traditional Chinese medicine Formula,Decoction or pills	Wu-Teng-Gao, Soufeng Sanjie Formula, Gancao Fuzi Decoction, Zishen Tongluo Formula, Xiaoying Daotan Decoction, Yanghe Decoction, Artemisinin and Hydroxychloroquine, BuYang HuanWu Decoction	Feiyanning Decoction, FuZheng FangAi Pill, Aiduqing Formula, Yi-Yi-Fu-Zi-Bai-Jiang-San (YYFZBJS), Shenling Baizhu Decoction (SLBZD), DaHuang ZheChong Pills, Modified Bu-Zhong-Yi-Qi Decoction, Jiawei Maxing Shigan Tang, Compound 511

It is noteworthy that some TCM compounds, such as Curcumin (Zeng et al., 2022; Liao et al., 2018; Yang et al., 2008) exhibit the ability to both promote and inhibit Treg cell proliferation and function. This is not a contradictory result, but rather a demonstration of their bidirectional regulatory role manifesting in different doses and disease backgrounds.

In summary, these results suggest that TCM mainly regulate Tregs proliferation and function by modulating *Foxp3* expression and the secretion of inhibitory cytokines.

### 4.3 Mechanisms of TCM in regulating Treg function

The specific mechanisms of action for different TCM metabolites are diverse. For instance, Gan Cao Fu Zi decoction (Zhao et al., 2023), total glucosides of paeony (Zhao et al., 2012), and oridonin (Guo et al., 2020), directly modulate *Foxp3* expression to regulate Treg function, while others achieve this by targeting Treg surface receptors. *CTD-1* promotes Treg differentiation and the secretion of inhibitory cytokines through AhR modulation (Shi et al., 2020), scutellarin (Chen S. et al., 2022) weakens Treg function by reducing *TNFR2* receptor expression; while berberine (Liu et al., 2020), luteolin (Jiang et al., 2021), and Modified Bu Zhong Yi Qi Decoction (Xu R. et al., 2020) diminish Treg suppressive function by downregulating *PD-1* receptor expression. Furthermore, Since *IL-2* stimulation is crucial for both Treg differentiation and maintenance of Treg function, the regulation of *IL-2R* is more important than that of other Treg surface receptors (Ono, 2020). This regulation can also be achieved through the modulation of the gut microbiota. Consequently, based on these distinct mechanisms, this review classifies TCM -mediated Treg regulatory mechanisms into four major categories: (1) Foxp3 expression regulation mechanisms; (2) IL-2 receptor pathway mechanisms; (3) Regulation of other Treg surface molecules; and (4) gut microbiota modulation mechanisms.

#### 4.3.1 *Foxp3* expression regulation mechanisms

The marker of T-cell differentiation into Tregs is the expression of *Foxp3,* and the regulation of Foxp3 expression can be achieved through various mechanisms. Studies have shown that the regulatory element region of the *Foxp3* locus contains a promoter and four conserved noncoding sequences (*CNS0-3*) (Kawakami et al., 2021; Bai et al., 2022). These cis-regulatory elements can be subject to DNA demethylation (Zong et al., 2021), histone modifications (Tao et al., 2007), and negative regulation by *miRNAs* (Yang L. P. et al., 2019), ensuring the stable transcription and expression of *Foxp3*. For instance, total glucosides of paeony promote the generation of Tregs by inducing *Foxp3* expression through reducing the DNA methylation level of the *Foxp3* promoter in lupus *CD4* T cells (Zhao et al., 2012). Gancao Fuzi decoction may regulate the imbalance of Th17/Tregs in rheumatoid arthritis by promoting *Foxp3* protein expression through the inhibition of *miR-34a* gene expression (Zhao et al., 2023).

However, how are these signals transmitted into the nucleus to achieve epigenetic regulation of the Foxp3 gene locus? Numerous studies suggest that TCMs transmit signals primarily through the *TGF-β/Smad* pathway and non-*TGF-β/Smad* pathways (Chen et al., 2003; Kanamori et al., 2016) (including the T-cell receptor (TCR) signaling pathway (Picca et al., 2006), and *NOTCH1* pathway (Asano et al., 2008)), ultimately promoting Treg differentiation.

##### 4.3.1.1 Classical pathway: *TGF-β/Smad* pathway

*TGF-β* plays an essential role in the transcription of Foxp3 and the generation of Tregs (Chen and Konkel, 2015). TGF-β binds to the *TGF-β* receptor (*TGF-βR)* on the surface of Tregs, activating Smad proteins. *CNS1* serves as a platform for *Smad2/3* molecules and induces *Foxp3* expression in a *TGF-β-*dependent manner, thus promoting the generation of Tregs (Sanjabi et al., 2017; Wang et al., 2023a). This signaling pathway is the classical pathway for inducing Treg differentiation. Research has indicated that at low concentrations, TGF-β synergizes with *IL-6* and *IL-21* to promote *IL-23R* expression, fostering the differentiation of T cells into Th17 cells. Conversely, at high concentrations, *TGF-β* inhibits *IL-23R* expression, promoting the differentiation of *Foxp3+* Tregs (Zhou et al., 2008). A study on mangiferin also demonstrated that adding MG, *TGF-β1*, or rapamycin to cell cultures significantly increased the percentage of *CD4+Foxp3+* Tregs (Liang et al., 2018). Oridonin inhibits *TGF-β1* signaling by promoting the degradation of the *TGF-βRI* and *TGF-βRII* proteins and reducing the phosphorylation of the *Smad2* and *Smad3* proteins. Through this pathway, it inhibits *Foxp3* expression, further suppressing Treg polarization (Guo et al., 2020). Researchers have used different polar solvents to extract DaHuang ZheChong Pill, a formula composed of 12 TCMs, into four polar fractions: water-soluble metabolites (PW), ethyl acetate (PE), n-butanol (PB), and petroleum ether (PP). Both PW and PE significantly inhibited Treg differentiation. PE reduces *TGF-β* mRNA and protein levels, and inhibits the phosphorylation of *Smad2* and *Smad3,* thus suppressing Treg differentiation. The water-soluble fraction (PW), on the other hand, primarily inhibits Treg differentiation by influencing hepatocellular carcinoma cell metabolism, improving TME acidity, and depleting glutamine (Wu et al., 2022). BYHWD also regulates Treg differentiation via this signaling pathway. *In vivo* experiments have demonstrated that BYHWD can upregulate the expression of *TGF-β, Smad2,* and *Foxp3* in peripheral blood, spleen, and aorta. *In vitro* experiments confirmed that BYHWD can reverse the inhibition of the *Foxp3/TGF-β/Smad2* pathway caused by blockers, thereby promoting Treg differentiation and improving atherosclerosis (Chen S. et al., 2021).

These findings suggest that TCM can modulate Treg generation by promoting or inhibiting *Foxp3* expression through the *TGF-β/Smad* signaling pathway.

##### 4.3.1.2 Non-classical pathway: T-cell receptor signaling and *Notch1* signaling

TCR signaling is a key pathway within the non-*TGF-β/Smad* signaling network. During thymic development, Tregs initiate a series of intracellular signaling events through high-affinity interactions between the TCR and self-antigen-MHC complexes, inducing Foxp3 expression without the requirement for *TGF-β* costimulation (Picca et al., 2006; Kim and Leonard, 2007). The intracellular regulators activated by TCR signaling can be categorized into two main groups: (1) Transcription factors: nuclear factor kappa-B(*NF-κB*), hypoxia-inducible factor-1α(*HIF-1α*), myelocytomatosis oncogene (*Myc*), nuclear factors of activated T cells (*NFAT*). (2) Metabolic kinases: phosphoinositide 3-kinase (*PI3K*), protein kinase B/(*Akt*)、AMP-activated protein kinase (*AMPK*) and mammalian target of rapamycin (*mTOR*). These regulators can directly regulate *Foxp3* expression (Park and Pan, 2015).

*NF-κB* is one of the key transcription factors activated by TCR signaling, and upon activation, it induces the classical *NF-κB* signaling pathway (Fulford et al., 2015). The *NF-κB* transcription factor family consists of five members: *c-Rel, p50 (NF-κB1), p52 (NF-κB2), p65 (RelA),* and *RelB.* The heterodimer of *p65 (RelA)* or *c-Rel* with *p50 (NF-κB1)* activates the classical *NF-κB* pathway, whereas the heterodimer formed by *RelB* and *p52 (NF-κB2)* activates the nonclassical pathway. *c-Rel* binds to the *CNS3* enhancer region, promoting *Foxp3* expression and thereby regulating Treg differentiation and function (Hövelmeyer et al., 2022; Oh et al., 2017; Long et al., 2009). The BuShen GuBiao Recipe can regulate Foxp3 expression via the *NF-κB* signaling pathway (Zhou et al., 2010), whereas the Ai Du Qing Formula can induce the differentiation of *CD4* ^*+*^ T cells into Tregs by activating the *NF-κB/Foxp3* pathway (Li et al., 2021). *Myc* and *HIF-1α* are transcription factors related to glucose metabolism that respond to TCR signaling (Chisolm and Weinmann, 2015). Ginkgo biloba extract promotes *Foxp3* expression and Treg differentiation by inhibiting *HIF-1α/HK2-*mediated glycolysis (Hui et al., 2023). TCR signaling activates the *PI3K/Akt/mTOR* pathway, which is typically considered a negative regulator. When excessively activated, it suppresses *Foxp3* expression, a topic that will be discussed in detail later.

*Notch1* signaling represents another crucial non-T*GF-β/Smad* pathway involved in regulating *Foxp3* transcription (Asano et al., 2008). Upon interaction between the Notch ligand and receptor, the Notch intracellular domain (*NIC)* is released into the cytoplasm. *NIC* then translocates into the nucleus, where it binds to the transcription factor RBP-J, forming an *NIC-RBP-J* complex that associates with the *Foxp3* promoter to regulate its expression. This pathway exhibits a biphasic regulation: low-intensity Notch signaling activates the *Foxp3* promoter via the *NICD-RBP-J* complex, whereas high-intensity signaling inhibits the promoter through *HES* (Ou-Yang et al., 2009; Vi et al., 2003). The TCM Xiaoying Daotan Tang and the levothyroxine sodium tablet (standard biomedical treatment) both effectively downregulate Notch1 protein expression. After treatment with Xiaoying Daotan Tang, the serum *TGF-β* levels in the mice increased significantly, whereas the *Foxp3* and *IL-10* levels did not. These finding indicate that Xiaoying Daotan Tang regulates the Treg/Th17 balance via the *Notch1* signaling pathway and enhances *TGF-β* expression (Zhou et al., 2021). Ganoderma polysaccharide extract induces the expression of micro*RNA-125b*, with Notch1 being a target of *miR-125b*. This interaction with the *Notch1* receptor, via Notch1 signaling, inhibits Foxp3 expression, reduces regulatory T cell accumulation, and suppresses the growth of hepatocellular carcinoma cells (Li et al., 2015). Resveratrol ultrafine nanoemulsion (*Res-mNE)* induces Treg differentiation by inhibiting the *Notch* signaling pathway and activating *Foxp3* expression, aiming to reverse the imbalance of Th17/Treg differentiation in immune thrombocytopenia (Cheng et al., 2023).

#### 4.3.2 *IL-2* receptor pathway mechanisms

The maintenance of Treg function requires *IL-2* stimulation. Tregs possess a high density of *IL-2* receptors (*IL-2R*), enabling them to competitively capture *IL-2* and thereby suppress the activation of effector T cells. Additionally, mature Tregs need continuous *IL-2* signaling to sustain their survival and suppressive function, which involves three major signaling axes via the *IL-2* receptor: the *STAT5, PI(3)K,* and *MAPK/ERK* pathways (Sakaguchi et al., 2008; Fan et al., 2018; Ross and Cantrell, 2018). Each of these pathways regulates the development and maintenance of Treg function (Zorn et al., 2006; Osinalde et al., 2011).

##### 4.3.2.1 *JAK/STAT5* signaling

Upon signal stimulation, *IL-2R* activates Janus kinase *(JAK*), which subsequently activates signal transducer and activator of transcription 5 (*STAT5)*. *STAT5* directly binds to the promoter and enhancer elements of *Foxp3*, inducing its expression and promoting Treg differentiation (Jones et al., 2020; Passerini et al., 2008). Wuwei Xiaodu Drink regulates *Foxp3* expression via the *IL-2/STAT5* signaling pathway (Huang K. et al., 2022), Bufei Yishen formula induces Treg differentiation by increasing *STAT5* phosphorylation levels, upregulating *Foxp3* gene expression, and correcting the Th17/Treg imbalance, which in turn improves lung function and alleviates inflammation in chronic obstructive pulmonary disease rats (Peng et al., 2018).

##### 4.3.2.2 *PI3K/Akt/mTOR* signaling

*PI3K* can be stimulated by various signals, activating the *PI3K/Akt/mTOR* pathway. When this pathway is activated via the *IL-2* receptor, it induces aerobic glycolysis in Tregs, and elevated glycolysis is detrimental to the stability and suppressive function of the Treg lineage (Fan and Turka, 2018). Moreover, sustained TCR signaling through this pathway also inhibits Foxp3 expression, however, early blockade of TCR stimulation can restore Foxp3 expression (Sauer et al., 2008). Therefore, this pathway negatively regulates *Foxp3* expression and Treg function (Luo and Li, 2013). Compound 511 reduces *Foxp3* mRNA levels and Treg generation by regulating the *PI3K/AKT/mTOR* signaling pathway (Li Z. et al., 2023). Glycyrrhiza, along with its active metabolites Gly1 and isoliquiritigenin, promotes Treg generation by attenuating the *TCR/Akt/mTOR* axis (Guo et al., 2015). Mangiferin induces *CD3* ^*+*^ T cell differentiation into *CD4+Foxp3+* Tregs and promotes Treg proliferation by inhibiting mTOR and the downstream phosphorylation of *P70S6K* (Liang et al., 2018).

##### 4.3.2.3 *MAPK/ERK* signaling

The mitogen-activated protein kinase (*MAPK*) pathway transmits signals from cell surface receptors to the DNA-binding protein chain in the nucleus (*Ras-Raf-MEK-ERK* pathway). It plays a regulatory role in processes such as cell proliferation and apoptosis (Gong et al., 2022; Bahar et al., 2023), and can also regulate Tregs. Dendrobium officinale national botanical drug drink reduces *Foxp3* expression and modulates the balance between Th17 and Tregs via the *SCFAs-GPR41/43-ERK1/2* pathway (Dong et al., 2024). Another study suggested that the *p38 MAPK* signaling pathway can activate *TNF*-mediated Treg proliferation, while SB203580, a *p38 MAPK* inhibitor, blocks LPS-induced Treg expansion and TNF expression in Tregs *in vivo*, thereby preventing *TNF*-mediated Treg proliferation (Chen and He, 2017). However, neither of these studies mentioned the involvement of *IL-2R* in the Treg process. Furthermore, this signaling pathway can be activated by *TCR*s, but the addition of exogenous *IL-2* stimulation during *TCR-CD3/CD28* stimulation significantly promotes Treg viability and expansion (Li et al., 2005). Moreover, in the *TGF-β* signaling pathway, inhibiting ERK activation enhances *TGF-β-*induced *Foxp3* expression and Treg development (Liu H. et al., 2013). These findings suggest that the *MAPK/ERK* pathway is not a typical pathway regulated via the *IL-2* receptor but rather acts as a bridge, integrating various regulatory pathways to modulate Tregs.

#### 4.3.3 Regulation of other Treg surface molecules

Some key signaling axes mediated by cell surface molecules also modulate Treg differentiation and function, with the *PD-1/PD-L1* regulatory axis being a prominent example (Gianchecchi and Fierabracci, 2018). For example, berberine specifically binds to CSN5 and inhibits its activity, destabilizing PD-L1 and preventing the activation of Tregs in the TME (69); modified Bu-Zhong-Yi-Qi Decoction inhibits tumor *PD-L1* expression via the *PI3K/AKT* pathway, while also reducing PD-1 expression on Tregs, thereby weakening their immunosuppressive effects (Xu R. et al., 2020); similarly curcumin lowers *PD-1* and *TIM-3* expression on Tregs, reducing their suppressive function, although the study has not explored the precise regulatory mechanisms involved (Liu et al., 2021). Thus, the *PD-1/PD-L1* axis, as a key regulatory axis for Treg suppressive function, can be modulated by various TCMs through different mechanisms. Many other similar cell surface molecules exist. For instance, scutellarin disrupts the *TNF-TNFR2* interaction, reducing *TNFR2* and *Foxp3* expression, thereby lowering the proportion of tumor-infiltrating Tregs (Chen S. et al., 2022). Cinnamtannin D1 promotes Treg differentiation by inhibiting *AhR* expression and upregulating *STAT5/Foxp3* (Shi et al., 2020).

While research on these cell surface molecule-mediated signaling pathways is not as systematic or extensive as that on the *Foxp3* and *IL-2R* signaling pathways mentioned earlier, it still provides new research avenues for TCM researchers. A major emerging perspective is the regulation of the gut microbiota.

#### 4.3.4 Gut microbiota modulation mechanisms

The gut microbiota plays a therapeutic role in various diseases, and TCM may also exert therapeutic effects by regulating the gut microbiome (Wei et al., 2024). Substantial research confirms that the regulatory effect of TCM botanical drugs and formulas on Treg cell differentiation is mediated by alterations to the gut microbiota. Multiple TCM botanical drug formulas have demonstrated this regulatory effect in animal models. For instance, Jiangu granule restores the abundance of the gut microbiota in rats, increase short-chain fatty acid content, reduce the permeability of the colonic epithelium to gut bacteria, increase the proportion of Tregs, and improve bone loss in ovariectomized rats (Sun et al., 2022). Similarly, Tongfu Lifei Decoction (Chen H. et al., 2024) and Modified Gegen Qinlian Decoction (Wang et al., 2021) were both observed to correct the Th17/Treg balance via gut microbiota modulation, alleviating sepsis-related intestinal mucosal injury and DSS-induced acute experimental colitis, respectively. Curcumin, a key metabolite from the botanical drug Curcuma longa L., likewise restored the Th17/Treg steady-state and upregulated the diversity and relative abundance of the gut microbiota in ulcerative colitis (UC) complicated by DM mice, effectively mitigating colitis in the Type 2 Diabetes Mellitus model (Xiao et al., 2022). Qingxie Fuzheng Granules (QFG) also rebalanced the Th17/Treg cell ratio through microbiota modulation in a cancer cachexia model (Jin et al., 2025). This collective evidence demonstrates that restoring the Th17/Treg balance remains a crucial checkpoint for various TCM botanical drugs to exert their therapeutic effects via the gut microbiota.

Focusing on this crucial checkpoint, researchers have also conducted in-depth mechanistic explorations. For example, studies on the anti-cachexia mechanism of QFG showed that it restored microbial balance by modulating dysbiosis (e.g., a decrease in Enterobacteriaceae and an increase in Lactobacillaceae). This action, coupled with the upregulation of tight junction proteins ZO-1, Occludin, and calprotectin, rebalanced the Th17/Treg cell ratio and inhibited *IL-6/NF-κB* signal transduction, thereby ameliorating cancer cachexia (Jin et al., 2025). This proposed mechanism effectively links the microbiota, intestinal barrier function, and inflammatory signaling pathways. Furthermore, Tuomin Dingchuan Decoction (Hong et al., 2025) was shown to promote Treg cell expansion via a Lactobacillus-dependent mechanism to alleviate asthma, underscoring the critical role of the *Lactobacillus* genus in Treg cell regulation. Shoutai pill (STP) (Xu et al., 2025) administration restores the gut microbial ecosystem and modulates the maternal Th17/Treg cell ratio through *JAK2/STAT3* signaling, thus stabilizing immune tolerance in early pregnancy. The therapeutic efficacy is attributed to metabolites chlorogenic acid, isochlorogenic acid A, and desmoside VI. Licorice water extraction (Sh et al., 2022) significantly improved the species and quantity of probiotics in the gut microbiota of UC mice. LWE reversed the Th17/Treg cell differentiation imbalance, and its mechanism was linked to changes in the colonic expression of *ROR-γt* and *Foxp3* proteins. Different therapeutic dosages of Chimonanthus nitens Oliv. Leaf Granules effectively improved the diversity and relative abundance of the gut microbiota in colitis mice. Specifically, Lachnospiraceae_NK4A136_group and Lachnospiraceae_UCG-006 were significantly enriched at the genus level, which correlated with Treg recruitment and the alleviation of oxidative stress damage (Huang J. Q. et al., 2022). The most direct mechanistic evidence comes from the research on fermented botanical drugs. Fermented Astragalus (FA), derived from the botanical drug Astragalus membranaceus (Fisch.) Bunge fermented with *Lactobacillus* plantarum, was analyzed to identify differential metabolites such as raffinose, progesterone, and uridine (11 in total). These metabolites were demonstrated to more effectively ameliorate DSS-induced colitis. Compared to unfermented Astragalus, FA-treated mice exhibited more pronounced gene expression of intestinal tight junction proteins (*ZO-1, Occlud*in) and mucin-secreting proteins (*MUC2*). Concurrently, pro-inflammatory factors (*TNF-*α*, IL-1*β*, IL-6, IL-17)* were downregulated, while anti-inflammatory factors (*IL-10, TGF-β)* were upregulated. This indicates that these metabolites intervene in the inflammatory state by modulating the balance of Th1/Th2/Th17/Treg-related cytokines (Li et al., 2022). Collectively, these mechanistic studies suggest that the *Lactobacillus* genus may participate in the regulatory process of Treg cells. However, the exact, molecule-level mechanisms by which the gut microbiota, or specific microbial metabolites, regulate Tregs still warrant further, more systematic exploration.

## 5 Conclusion, limitation and prospect

### 5.1 Conclusion

This review highlights the distinctive immunoregulatory effect of TCM on Tregs, which can manifest as either immune-enhancing or immune-suppressive effects depending on the host’s pathological state. This characteristic reflects the fundamental TCM concept of “*Fuzheng Quxie*” (supporting the upright and dispelling the evil) and “*Balancing Yin-Yang,*” where the ultimate goal is to restore immune homeostasis rather than induce unidirectional stimulation or suppression. It should be emphasized that this “bidirectional regulation” represents a context-based hypothetical concept rather than a fully established molecular mechanism. Current evidence suggests that disease microenvironment, cytokine milieu, and pharmacological exposure may collectively determine the direction of Treg modulation, yet the key molecular determinants underlying this “directional switching” remain to be elucidated. Future mechanistic and systems-level studies are warranted to validate this hypothesis and identify the critical signaling pathways that mediate the context-dependent immunoregulatory effects of TCM.

**FIGURE 1:**
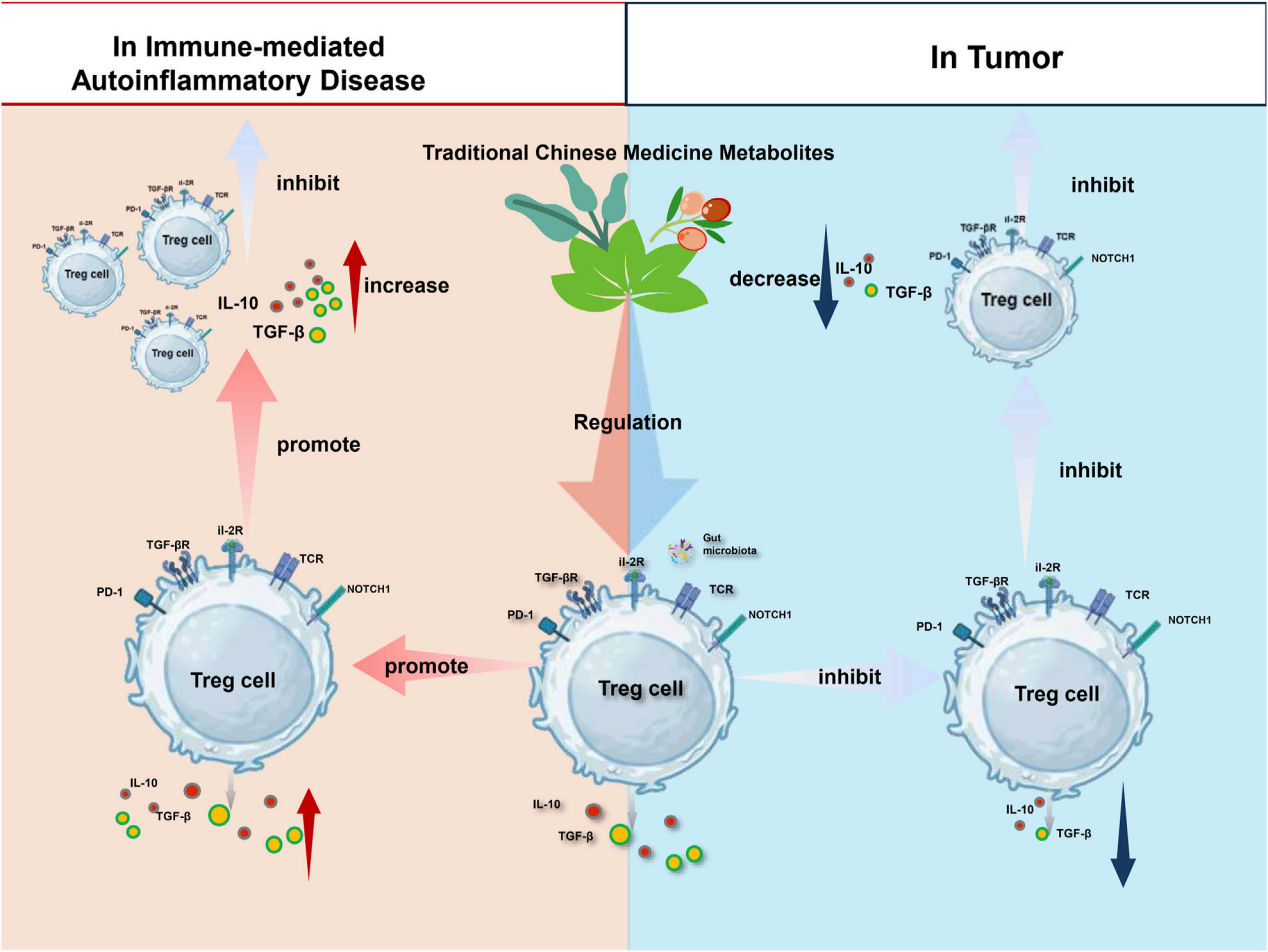
Regulation of Treg Homeostasis by TCM Metabolites in Disease Contexts Traditional Chinese Medicines (TCMs) exhibit a regulatory effect on Treg cells through various pathways. By enhancing the expression of Foxp3, TCMs promote Treg cell differentiation and the secretion of inhibitory cytokines, thereby contributing to the treatment of immune-related autoimmune inflammation. Conversely, by suppressing Foxp3 expression, TCMs inhibit Treg cell differentiation and the release of inhibitory cytokines, thereby counteracting tumor immune evasion and restraining tumor growth. Created in BioRender.

Therefore, the “bidirectional regulation” should be regarded as an overarching characteristic of TCM’s holistic immunomodulation, rather than the property of a single compound. Several representative metabolites further exemplify this context-dependent characteristic. For instance, Curcumin and Glycyrrhizin (from Glycyrrhiza uralensis) display opposite effects on Tregs across different disease backgrounds: in autoimmune or inflammatory conditions, they can promote Treg differentiation and *Foxp3* expression to restore immune tolerance; whereas in tumor models, they often suppress Treg accumulation in the tumor microenvironment, thereby activating anti-tumor immunity. This difference is not a contradiction but rather reflects the environment-dependent nature and flexibility of TCM’s immunomodulatory action. Understanding this bidirectionality is essential for the rational application of TCM based on specific disease characteristics, enabling the precise regulation of immune balance for optimal therapeutic outcomes (Figure 1).

**FIGURE 2:**
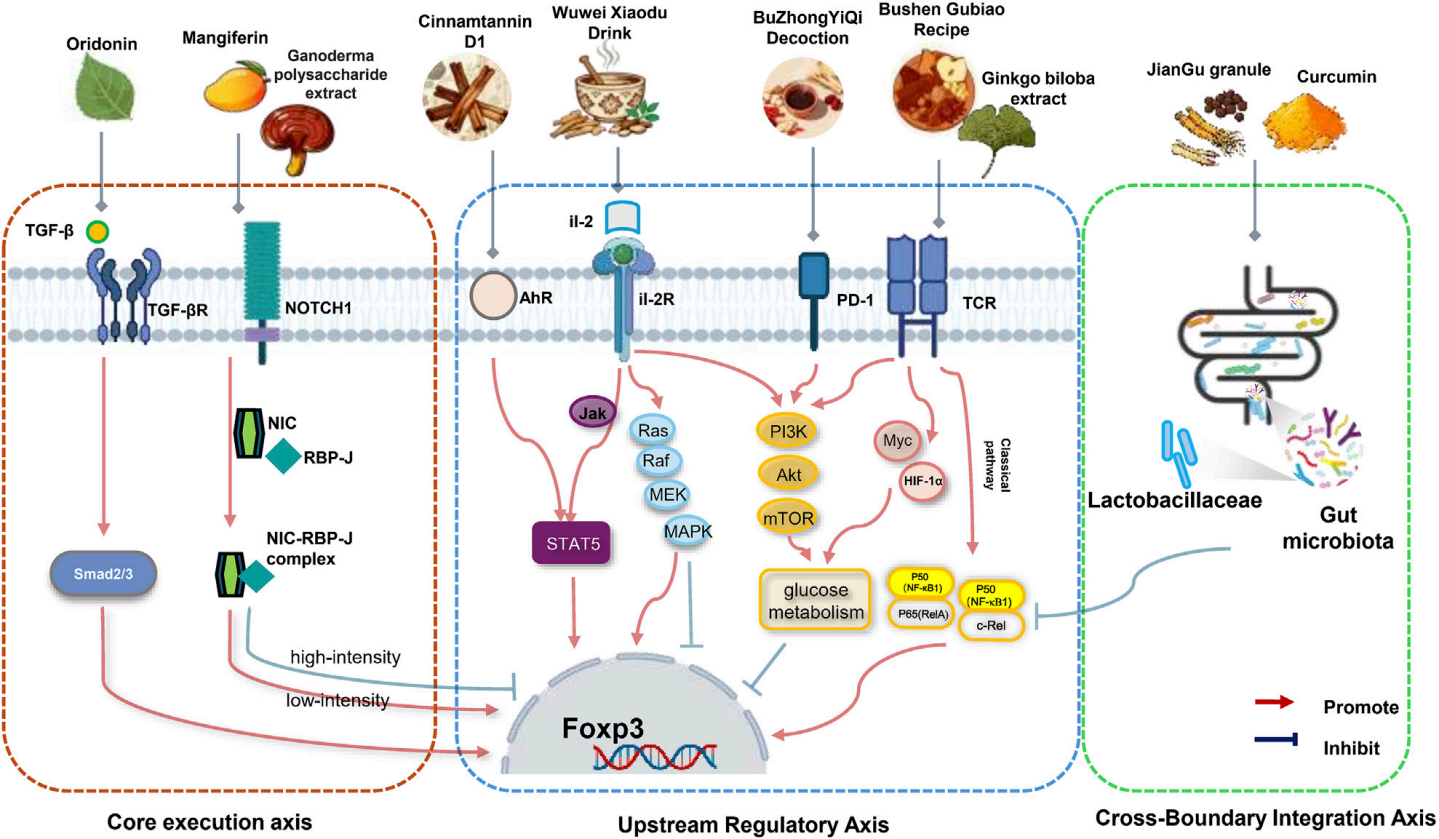
Integrative Quad-Mechanistic Framework of TCM Treg Regulation via Core, Upstream, and Cross-Boundary Axes Mechanisms of TCM in regulating Treg cell. This figure illustrates the comprehensive hierarchical quad-mechanistic framework through which Traditional Chinese Medicine (TCM) botanical drugs and their metabolites modulate regulatory T (Treg) cell differentiation and function via three integrated and interactive axes: (1) Core Execution Axis (Direct molecular pathways controlling Foxp3): Classical pathway: TGF-β binds to its receptor (TGF-βR), activating Smad2/3 signaling, a primary inducer of Foxp3 expression. (e.g., Oridonin). Non-classical pathway: Notch1 signaling, where Notch intracellular domain (NIC) forms a complex with RBP-J. Low-intensity NIC-RBP-J promotes Foxp3, while high-intensity signaling inhibits it. (e.g., Mangiferin) (2) Upstream Regulatory Axis (Surface molecule crosstalk): Interactions involving PD-1and AhR affect Treg expansion. (e.g., Cinnamtannin D1, BuZhongYiQi Decoction). IL-2R signaling: IL-2 binding to IL-2R activates STAT5, crucial for Treg survival and suppressive function(e.g.,Wuwei Xiaodu Drink ), Inflammatory and metabolic pathway modulation: Signaling cascades PI3K/Akt/mTOR, and Ras/Raf/MEK/MAPK regulate Foxp3 by influencing glucose metabolism and inflammatory tone. TCM formulas (e.g., Bushen Gubiao Recipe, Ginkgo biloba extract) (3) Cross-Boundary Integration Axis (Gut microbiota): Microbial enrichment and metabolite production promote beneficial gut bacteria (e.g., Lactobacillaceae). Microbiota-immune crosstalk: Microbial products interact with host signaling (e.g., via NF-κB subunits p50 and p65/c-Rel) to regulate Foxp3, linking microbial metabolism to Treg-mediated immune tolerance: TCMs (e.g., JianGu granule, Curcumin). Created in BioRender.

The central contribution of this review is the systematic integration of the core mechanisms by which TCM botanical drugs and their metabolites modulate Treg cells, culminating in the proposal of a hierarchical four-dimensional mechanistic framework encompassing master transcription factors, *IL-2* receptor pathways, other surface molecules, and the gut microbiota. This study strongly confirms that correcting the imbalance of the Th17/Treg ratio is the primary objective of various TCMs. This goal is achieved through a multi-layered intervention on Treg-targeted pathways, which can be summarized into three interconnected axes (Figure 2): (1) Core execution axis: Multi-Pathway Integration for *Foxp3* Expression Regulation. This axis represents the most direct and primary molecular mechanism by which TCMs regulate Tregs. It involves the precise control of the master transcription factor *Foxp3* through a multi-pathway approach, including both classical and non-classical signaling pathways. Whether TCMs induce Foxp3 expression through the typical *TGF-β/Smad* pathway (Chen and Konkel, 2015) or precisely modulate Foxp3 expression by intervening in the non-classic`al TCR (Picca et al., 2006), (Fulford et al., 2015) and Notch1 signaling pathways (Asano et al., 2008), this represents the most direct and primary action of TCMs in regulating Tregs. (2) Upstream regulatory axis: Crosstalk between surface molecules/inflammatory pathways and signaling pathways. This axis demonstrates how TCMs indirectly influence *Foxp3* expression by modulating the cellular microenvironment and intercellular signals. These signaling pathways do not function in isolation but exhibit significant crosstalk. For example, the *PD-1/PD-L1* axis, a surface molecule, participates in Treg cell proliferation by modulating the *Notch* pathway (Cai et al., 2019). Similarly, *Notch1* signaling can enhance the effector function of *TGF-β*-mediated Tregs, as evidenced by the treatment of Hashimoto’s thyroiditis with Xiaoying Daotan Tang (Zhou et al., 2021). This crosstalk suggests that TCMs can indirectly affect *Foxp3* expression and Treg function by regulating surface molecules like *PD-1*. Furthermore, the crosstalk between hyperactivated *Notch3* and the classical *NF-κB* pathway upregulates *Foxp3* expression, thereby enhancing the ability of Tregs to suppress protective anti-tumor immune responses within the tumor microenvironment (TME) (Ferrandino et al., 2018). Likewise, the inhibition of inflammatory pathways (*NF-κB* and *JAK/STAT*) creates and maintains a low-inflammatory microenvironment for Treg cells, acting as a synergistic effect. In addition, the I*L-2R/STAT5* axis (Huang K. et al., 2022), as a core pathway for maintaining Treg function, is also an important target for TCMs (e.g., Bufei Yishen formula (Peng et al., 2018)). (3)Cross-boundary integration axis: Gut microbiota modulation of immune pathways. The regulatory effect of TCMs on Tregs is achieved by altering the structure and function of the gut microbiota (Wei et al., 2024), establishing a crucial cross-boundary mechanism within the quad-mechanistic framework. Existing research clearly reveals a complex chain of events by which TCMs modulate Tregs via the gut microbiota:1. Microbial effects and metabolic mediation: This mechanism involves TCM metabolites → promoting the abundance of specific probiotics (e.g., *Lactobacillus* (Hong et al., 2025; Li et al., 2022)) → increasing the concentration of microbial metabolites like short-chain fatty acids (SCFAs) (Dong et al., 2024) → acting on host receptors → regulating *Foxp3* expression. Notably, SCFAs act on host cell surface GPRs receptors, serving as a key messenger connecting gut microbial metabolism with Treg differentiation. Microbial product-mediated *TLR4/NF-κB* Crosstalk: The regulation of the gut microbiota by TCMs essentially involves controlling the stimulation of host cells by microbial products. For instance, *E. coli* and its products can activate the *TLR4/NF-κB* signaling pathway, leading to a Th17/Treg imbalance. This suggests that TCMs, by improving the gut microbial structure, reduce the activation of the *TLR4/NF-κB* axis by microbial-associated molecular patterns (MAMPs), thereby synergistically maintaining Treg-mediated immune tolerance (Wang et al., 2023b). Synergistic Action on Intestinal Barrier: The ability of TCMs (e.g., QFG (155), FA (Li et al., 2022)) to repair intestinal tight junction proteins (upregulating *ZO-1* and *Occludin)* is critical for reducing inflammatory stimulation. This maintenance of intestinal barrier function provides a stable physiological microenvironment for Treg cells, serving as an important synergistic foundation for sustaining their immunosuppressive activity.

### 5.2 Limitation

Despite this review systematically integrating the mechanisms of TCM regulating Tregs and proposing a hierarchical framework, we must acknowledge the following three inherent limitations of both current research and this review itself. (1) Weak causality chain in mechanistic validation: Most mechanistic findings in the existing literature remain at the level of correlation, lacking crucial causal validation. Specifically regarding this review’s core innovation—the Cross-boundary integration axis (Gut Microbiota-Treg axis)—studies generally lack functional intervention and verification using gold-standard methods (e.g., fecal microbiota transplantation), leaving the causal link between TCM-mediated microbial modulation and Treg regulation incomplete. (2) Metabolite complexity and insufficient quantitative analysis: Current mechanistic studies face the challenge of TCM’s high metabolite complexity, making it difficult to pinpoint which specific metabolites exert the dominant effects *in vivo*. Concurrently, research often relies on the analysis of single signaling pathways, lacking the use of multi-omics technologies (e.g., metabolomics, macro-genomics) for the systematic, quantitative resolution of the complex regulatory network. (3) Lack of pharmacokinetic data and clinical translation evidence: The majority of studies fail to fully explore the pharmacokinetics, ADME (absorption, distribution, metabolism, and excretion), and bioavailability of TCM metabolites or their metabolites at target tissues, hindering a complete understanding of the mechanism. More critically, as this review is predominantly based on cell and animal models, there is a scarcity of multicenter, high-quality Randomized Controlled Trials to fully validate the actual efficacy, safety, and dose dependency of TCM-mediated Treg regulation in human disease treatment.

### 5.3 Prospect

The application of TCM still faces significant challenges due to the chemical diversity and mechanistic complexity of its metabolites. Overcoming these hurdles requires advanced techniques to fully elucidate metabolite-specific effects and safety. To advance TCM-based immunotherapies, future research must prioritize: (1) Targeted discovery and mechanism elucidation of novel metabolites: Focusing on isolating novel, high-potency metabolites and precisely clarifying their specific mechanisms in regulating Tregs to establish a groundwork for drug development. (2) Validation of causal links and quantitative network analysis: Moving beyond correlation by applying gold-standard methods (e.g., fecal microbiota transplantation, sterile mouse models) to validate the causal link within the gut microbiota-Treg axis, alongside utilizing multi-omics technologies for the systematic, quantitative resolution of the complex Treg regulatory network. (3) Synergistic Studies with Immunotherapy and Clinical Translation: Exploring TCM’s synergy with existing immunotherapies (e.g., immune checkpoint inhibitors) to enhance efficacy and reduce side effects by balancing the Treg/Th17 ratio, thus positioning TCM as a safer key metabolite in comprehensive treatment regimens.
