# Supplementary material for: Pharmacological mechanisms of traditional Chinese medicine metabolites in regulating Treg cells: an integrative pathway review
Source: Front Pharmacol. 2025 Dec 11;16:1527421. doi: 10.3389/fphar.2025.1527421 (PMC12738957; doi:10.3389/fphar.2025.1527421)
Supplement: Supplementary file 1 [file Table1.docx]

| Rigor Assessment | Reference NO. |
| --- | --- |
| High Rigor | 37-47,50-66,70,71,74,77,79-101,111-114,119,145,146,151-159 |
| Moderate Rigor | 48,49,67,68,69,72,73,75,76,78 |
| Low Rigor | 65 |

*Supplementary Tables 1*
